# Negative log-binomial model with optimal robust variance to estimate the prevalence ratio, in cross-sectional population studies

**DOI:** 10.1186/s12874-023-01999-1

**Published:** 2023-10-04

**Authors:** Milcíades Ibáñez-Pinilla, Sara Villalba-Niño, Nury N. Olaya-Galán

**Affiliations:** 1 Escuela de Medicina Y Ciencias de La Salud, Universidad del Rosario, Bogotá, Colombia; 2 Facultad de Medicina, Fundación Universitaria Sanitas, Bogotá, Colombia; 3 Mederi Research Center, Mayor Mederi University Hospital, Bogotá, Colombia; 4 Centro de Investigación en Salud, Universidad San Martín, Bogotá, D.C Colombia; 5 UniSabana Center for Translational Science (UCTS), Universidad de La Sabana, Chía, Colombia

**Keywords:** Cross-Sectional Studies, Prevalence Ratio, Logistic Models, Odds Ratio, Maximum Likelihood Estimation and Binomial Distribution

## Abstract

**Background:**

Cross-sectional studies are useful for the estimation of prevalence of a particular event with concerns in specific populations, as in the case of diseases or other public health interests. Most of these studies have been carried out with binary binomial logistic regression model which estimates OR values that could be overestimated due to the adjustment of the model. Thus, the selection of the best multivariate model for cross-sectional studies is a priority to control the overestimation of the associations.

**Methods:**

We compared the precision of the estimates of the prevalence ratio (PR) of the negative Log-binomial model (NLB) with Mantel–Haenszel (MH) and the regression models Cox, Log-Poisson, Log-binomial, and the OR of the binary logistic regression in population-based cross-sectional studies. The prevalence from a previous cross-sectional study carried out in Colombia about the association of mental health disorders with the consumption of psychoactive substances (e.g., cocaine, marijuana, cigarette, alcohol and risk of consumption of psychoactive substances) were used. The precision of the point estimates of the PR was evaluated for the NLB model with robust variance estimates, controlled with confounding variables, and confidence interval of 95%.

**Results:**

The NLB model adjusted with robust variance showed accuracy in the measurements of crude PRs, standard errors of estimate and its corresponding confidence intervals (95%CI) as well as a high precision of the PR estimate and standard errors of estimate after the adjustment of the model by grouped age compared with the MH PR estimate.

Obtained PRs and 95%CI entre NLB y MH were: cocaine consumption (2.931,IC95%: 0.723–11.889 vs. 2.913, IC95%: 0.786–12.845), marijuana consumption (3.444, IC95%: 1.856–6.391 vs. 3.407, IC95%: 1.848, 6.281), cigarette smoking (2.175,IC95%: 1.493, 3.167 vs. 2.209, IC95%: 1.518–3.214), alcohol consumption (1.243,IC95%: 1.158–1.334 vs. 1.241, IC95%: 1.157–1.332), and risk of consumption of psychoactive substances (1.086, IC95%: 1.047–1.127 vs. 1.086, IC95%: 1.047, 1.126). The NLB model adjusted with robust variance showed mayor precision when increasing the prevalence, then the other models with robust variance with respect to MH.

**Conclusions:**

The NLB model with robust variance was shown as a powerful strategy for the estimation of PRs for cross-sectional population-based studies, as high precision levels were identified for point estimators, standard errors of estimate and its corresponding confidence intervals, after the adjustment of confounding variables. In addition, it does not represent convergence issues for high prevalence cases (as it occur with the Log-binomial model) and could be considered in cases of overdispersion and with greater precision and goodness of fit than the other models with robust variance, as it was shown with the data set of the cross-sectional study used in here.

## Background

The objective of cross-sectional population-based research is to estimate prevalence of diseases or other events of interest for the human public health such as mortality, rehospitalization, quality of life, psychoactive substances consumption, among some others, and their associated (exposure) factors of these events of interest. Cross-sectional studies at the population level are usually designed with probabilistic samples with random selection, inferred or representative of the study population giving as a result unbiased, efficient, consistent, and sufficient estimators of prevalence and its corresponding 95% confidence intervals, with the estimation of standard and relative standard errors as precision indicators of the point estimates of the prevalence of the events of interest [1, 2].

In addition, in the cross-sectional studies as there is a construction of explanatory factors of the disease or the event, it should be an adjustment of the confounding factors that could lead to misinterpretations of the results if it is not well adjusted, considering individual factors but also its possible interactions among the variables of the model [3, 4]. For cross-sectional studies, binary logistic regression model has been used as a common and frequent strategy for the estimation of *odds ratio* (OR) and 95%CI as a measurement for the association of the outcome (disease or event) with potential explanatory variables. However, OR value overestimates the associations regarding to the prevalence ratios (PR = p1/p2) estimates, with an increased bias in the case of diseases or events with high prevalence [3–5].

Since 2003, Cox constant time model, Log-Poisson model, and Log-binomial regression model were proposed for controlling the bias of the overestimation of OR values in the case of binary logistic regression models, giving as a result good accuracies for the estimation of prevalence ratios (PR), although it should be evaluated the mathematical assumptions to validate these models [3, 4]. In the Poisson model one of the assumptions is the equality between the expected value and the estimated variance, in which is frequent to obtain greater variance values compared with the expected values, generating overdispersion of the data (extra-Poisson variance) in the model [3]. The overdispersion of the data leads to the underestimation of the standard error coefficients, deriving significant associations of explanatory factors of the event of interest which, do not exist [6]. In the case of the Log-binomial regression model, it has convergence issues when comparing numerical covariables as well as in the cases of high prevalence of the diseases or events [3, 4]. Finally, in the Cox model it is frequent the non-fulfillment of the assumption of proportional risks in the population at a time *t* of the observation, which is adjusted considering a constant time for the estimation of the prevalence ratios in the cross-sectional studies [3, 4].

Thus, although the above-mentioned models have been used in the research field for the estimation of PRs in cross-sectional studies, there are still some issues in the models that could be improved. The negative Log-binomial regression model (NLB) has shown high mathematical consistency in the application of longitudinal cohort analytical studies and has been used in cases of overdispersion of the Poisson model [6–10]. NLB is proposed in this study as a novel generalized linear model for the estimation of prevalence ratios (PR), as a measure of association and control for confounding categorical and numerical variables in cross-sectional population-based studies as a strategy for controlling the bias obtained in unconditional binary logistic regression models due to the overestimation of OR values. In this study, a comparison of the NLB model with the Mantel–Haenszel (MH) stratification method and the three current models for the estimation of PRs is proposed, using a previous study carried out in Colombia about the consumption of psychoactive substances and mental disorders as a precision and accuracy indicator for the estimates of the PR using the NLB model.

## Methods

### Study description

The data used to compare the point and interval estimates of the models of the present study were taken from a previous cross-sectional population-based study, with the specific objective of estimating the prevalence of psychoactive substances consumption and its associated factors in a population of 140,000 workers in Colombia, in which a stratified random probability sample of 5810 workers was selected.

The outcomes or events of interest in this study were: lifetime cocaine use, marijuana consumption in the last year, current cigarette consumption, lifetime alcohol consumption risk of consumption of psychoactive substances. The association factor used in the model was depression measured with the Zung test and grouped ages were used as a confounding variable with the following categories: 1) under 25, 2) between 26 and 29, 3) between 30 and 34, and 5) older than and equal to 35 years. It was used as a numerical variable as well.

### Negative Log-binomial regression model (NLB)

For the use of the negative Log-binomial regression model, it is important to keep in mind that the probability distribution of the negative binomial discrete random variable X measures the number of trials necessary to obtain r-successes, with independent trials and its parameters are *r* (total number of successes) and *p* which is the probability of success, therefore the probability of failure is q = 1-p and the negative binomial probability distribution is as follows (Eq. 1):

1 $${b}^{*}\left(x;r,p\right)=\left(\begin{array}{c}x-1\\ r-1\end{array}\right){p}^{r}{q}^{x-r}, x=r+r+1+r+2\dots \dots \dots$$

This Poisson-derived distribution, adjusted for overdispersion, has an expected value different from the variance, as shown below (Eqs. 2 and 3):

2 $$E\left(X\right)=\frac{r\left(1-p\right)}{p}$$

3 $$Var\left(X\right)=\frac{r\left(1-p\right)}{{p}^{2}}= \frac{1}{p}E\left(X\right)$$

In the construction of the generalized linear model (GLM) of the proposed negative log-binomial model, the three components of the GLM were taken into account: in the random component, the random variable of the dependent variable of the negative binomial model (Y), with exponential family distribution is measured in counts occurring at a time *t* and is also used in continuous and dichotomous variables evaluating the mathematical assumptions of the model (linearity of the model parameters and independence of the observations of the study subjects), therefore it is applicable in cross-sectional studies in the estimation of the prevalence of the disease or dichotomous variables of cross-sectional studies. The systematic components of the model which are the explanatory variables or associated factors *Xi*, numerical or categorical variables with their respective estimators (*β*_*i*_), standard errors and confidence intervals, which are used for the construction of the associative models of the cross-sectional studies. Finally, for the link function, in this case was the logarithm per se, which was proposed the name of "negative log-binomial" model for cross-sectional studies.

Maximum likelihood was used as the estimation method of the NLB and to compare iteratively reweighted least squares (IRLS), with Fisher scoring, Newton–Raphson and hybrid iterative optimization methods for convergence tolerances estimations or epsilon (ε) < 0.000001 or 1e-6 (ε > 0).

NLB model is derived from a compound Poisson distribution with fitted Gamma distribution [8], with the log link *g(μ)* = *ln(μ)=Xi βi*,  vi=1/α, then  Yi⁄Xi =BN(1/α,1/(1+αμi) )as follows (Eq. 4):

4 $$lh\left({}^{{Y}_{i}}\!\left/ \!{}_{{X}_{i}}\right.\right)={\widehat{\beta }}_{0}+{\widehat{\beta }}_{1}{X}_{1}+{\widehat{\beta }}_{2}{X}_{2}+\dots .+{\widehat{\beta }}_{k}{X}_{k}$$

In the negative log-binomial model taking as independent variable dummy *X*_*i*_, with the values 0 and 1 (k = 0 and k = 1), the incidence rate ratio (IRR) of the binomial model was taken as the relative risk in analytical cohort studies and thus as the prevalence ratio (PR) in cross-sectional studies, as follows (Eqs. 5 and 6) [8–10]:

5 $$IRR=\frac{{{e}^{{\widehat{\beta }}_{i}\left(x+k\right)}}}{{e}^{{\widehat{\beta }}_{i}\left(x\right)}}={e}^{{\widehat{\beta }}_{i}\left(x+k\right)-{\widehat{\beta }}_{i}x}={e}^{\widehat{{\beta }_{i}}k}=PR$$

With k = 1, in cross-sectional studies, the RP estimator as follow:

6 $${e}^{{\widehat{\beta }}_{i}}=\widehat{PR}$$

### The inherent bias of the OR versus the PR

The inherent bias of the OR versus the PR for the NLB model was calculated to estimate the PRs for the five outcomes for the Colombian study.

In analytical epidemiological studies, it has been shown that the *odds ratio* (OR) calculation is an accurate estimator of the relative risk (RR) for the cases in which the prevalence of the disease is small (*p* < 10%) and therefore for the prevalence ratio in cross-sectional studies [5–10]. The PR is measured as the proportion of the prevalence of individuals with disease exposed to a specific factor over the proportion of the prevalence of individuals with disease without exposure (PR = p1/p2). The *odds ratio* is defined as a ratio of odds (odds = prevalence/(1-prevalence) = p/q = p/1-p) [7] and the calculation of the OR in cross-sectional studies is the odds of disease in exposed compared to the odds of disease in unexposed [7].

In a cross-sectional study the prevalence of the disease are taken, in the category of exposure *p*_*1*_ and in the category without exposure *p*_*2*_, when evaluating the association with higher prevalence in the exposed than in the non-exposed (risk), it could be seen that ${p}_{2}<{p}_{1}$, thus ${(1-p}_{2})> {(1-p}_{1})$ and in the case of protector factor, ${p}_{1}<{p}_{2}$, thus ${(1-p}_{2})< {(1-p}_{1})$ , which is the inherent bias of OR versus PR, as shown in Eqs. 7 and 8  [7].

7 $$OR=\frac{{}^{{p}_{1}}\!\left/ \!{}_{{q}_{1}}\right.}{{}^{{p}_{2}}\!\left/ \!{}_{{q}_{2}}\right.}=\frac{{}^{{p}_{1}}\!\left/ \!{}_{{(1-p}_{1})}\right.}{{}^{{p}_{2}}\!\left/ \!{}_{{(1-p}_{2})}\right.}=\frac{{p}_{1}}{{p}_{2}}x\frac{{(1-p}_{2})}{{(1-p}_{1})}=PRx\frac{{(1-{\varvec{p}}}_{2})}{{(1-{\varvec{p}}}_{1})}$$

8 $$\frac{{(1-p}_{2})}{{(1-p}_{1})} Represents inherent bias that OR has respecting to PR$$

This inherent bias is controlled when the prevalence ${p}_{1}$ and ${p}_{2}$ belong to small values (*p* < 10%) and increases with higher prevalence of the disease or event of interest [7].

### Estimator precision and confusion equations

Crude prevalence ratios (PR) and standard errors of estimation were considered as the reference measures of the associations (2 × 2 tables) in conjunction with the Mantel–Haenszel (MH) stratification method to control confounding variables.

The precision of the PR estimates of the regression models was measured compared with PR MH reference standard, as described in equation

9 $$Precision of the PR of the models, with respect to PR DE MH (\%)=\frac{\left|PR MH - PR Model \right|}{PR Model }*100$$

The indicator of the percentage of confounding effect between the association of depression with the outcomes of legal and illegal psychoactive substances consumption was also measured controlling for the grouped age confounding factor, for both crude PR of the NLB and the other three models, and the reference PR value of MH model as indicated in Eq. 10.

10 $$Confusion percentage=\frac{\left(crude PR -MH PR or from the model\right)}{MH PR or from the model}*100$$

This equation is also used to compare the standard error of estimation of the models with respect to the standard error of MH.

11 $$Precision of the standard error of the RP of the models, with respect to the standard error of MH (\%)=\frac{\left|standard error of estimate of MH PR-Model error estandar de estimaci\acute{o} n of PR\right|}{Model error estandar de estimaci\acute{o} n of PR }*100$$

The PR, standard errors of estimation and 95% confidence intervals with and without robust variance adjustment of the NLB model were compared with the three models (Cox time-constant, Log-Poisson and Log binomial) and with the unconditional logistic regression model with and without robust variance adjusted by age, with both grouped and numerical variable. Estimations were performed in STATA 15.0 [11] and SPSS version 25.0 [12]. The BIC Bayesian criterion is also used to select the best model.

## Results

### Cross-sectional study description

In the cross-sectional study of mental health and psychoactive substance consumption, a probabilistic, stratified random sample with proportional allocation was designed in 5810 workers in Colombia. The age of the workers varied between 18 and 56 years, with an average of 28.2 ± 7.1 years (median = 27.0 years) and age groups of ≤ 25 years (41.9%), 26 to 29 years (24.0%), 30 to 34 years (14.0%) and 35 and over (20.2%), with a predominance of male gender (93.4%), single marital status (48.8%), followed by married (30.0%).

In this cross-sectional study the consumption of psychoactive substances was measured, estimating a lifetime prevalence of cocaine consumption of 1.8% CI 95% (1.4% -2.1%), prevalence of marijuana consumption of 9.6% CI 95%:(8.8%- 10.3%), prevalence of cigarette consumption of 21.3% CI 95%: (20.1%- 22.4%), lifetime prevalence of alcohol intake of 85.7% CI 95%:(84.8.0%- 86.6%) and risk of consumption of psychoactive substances of 96.1% CI 95%: (95.6%- 96.6%).

The inherent bias of crude OR versus crude PR was increased the overestimation by higher prevalence of consumptions; for cocaine 1.2%, marijuana 7.9%, cigarette 17.5%, alcohol 133.3% and risk of consumption of psychoactive substances 233.3%, the overestimation of the association of the OR versus the PR being very high in the last two cases due to their high prevalence. The OR and PR estimators of association were different in the 5 outcomes, the overestimation of the OR being greater as the prevalence increases (Table 1).

**Table 1:** Prevalence rates ratio crudes and of the NLB model of the consumption of cocaine, marijuana, cigarette, alcohol risk of consumption of psychoactive substances associated with depression of Colombian workers

			CI 95%	
	**Estimator**	**Standard error estimation**	**Lower limit**	**Upper limit**
**Lifetime cocaine consumption prevalence (prevalence = 1.8%)**				
PR Crude	3.294	0,7126	0.815	13.316
PR Negative Log-Binomial model	3.294	0.7168	0.809	13.424
PR Robust Negative Log-Binomial model	3.294	0.7126	0.815	13.316
OR Crude – logistic regression binary	3.337	0.717	0.819	13.600
**Marijuana consumption prevalence (prevalence = 9.6%)**				
PR Crude	3.493	0,2999	1.940	6.287
PR Negative Log-Binomial model	3.493	0.3091	1.906	6.401
PR Robust Negative Log-Binomial model	3.493	0.2999	1.940	6.287
OR Crude – logistic regression binary	3.771	0.309	2.057	6.913
**Cigarette consumption prevalence (prevalence = 21.3%)**				
PR Crude	2.593	0.1897	1.788	3.760
PR Negative Log-Binomial model	2.593	0.2073	1.727	3.893
PR Robust Negative Log-Binomial Model	2.593	0.1897	1.788	3.760
OR Crude -logistic regression binary	3.045	0.208	2.025	4.579
**Lifetime alcohol consumption prevalence,(prevalence = 85.7%)**				
PR Crude	1.255	0,0356	1.171	1.346
PR Negative Log-Binomial model	1.255	0.0849	1.063	1.463
PR Robust Negative Log-Binomial model	*1.255*	*0.0356*	*1.171*	*1.346*
OR Crude – logistic regression binary	2.940	0.121	2.319	3.729
**Risk of consumption of psychoactive substances (prevalence = 96.1%)**				
PR Crude	1.086	0.0180	1.048	1.125
PR Negative Log-Binomial model	1.086	0.0769	0.934	1.263
PR Robust Negative Log-Binomial model	1.086	0.0180	1.048	1.125
OR Crude – logistic regression binary	3.560	0.180	2.501	5.068

Significant associations were identified between depressive symptoms with marijuana, cigarette, alcohol use and risk of consumption of psychoactive substances and close to significant differences with cocaine consumption, in the bivariate and multivariate analysis with MH (Table 1 y 2).

The PR, standard errors and 95% CI of the NLB model with robust variance were exactly equal to the raw values (Table 1). In the Cox and Poisson models with robust variance, the same results as NLB were found, although in the binomial regression the difference was in the risk of consuming psychoactive substances, which did not show convergence.

### Comparison of the estimation of the PR and its precision with the current models: Cox model with constant time, Log-Poisson, and Log-binomial

Comparison of the estimation of the PR and its precision of the Cox models with constant time, Log-Poisson, and Log-binomial with adjustment for confounding variables was included considering MH as reference. The results of the association of depressive symptoms with psychoactive substance consumption, controlled for the confounding factor of worker age, were like those obtained in the bivariate analysis, when controlling for age, adjusting for a confounding effect, the confounding effects of the models with respect to MH were similar (Table 2).

**Table 2:** Prevalence rates ratios and 95% confidence intervals of the consumption of cocaine, marijuana, cigarette, alcohol risk of consumption of psychoactive substances associated with depression adjusted for age groups of Colombian workers

				CI 95%			
	**Estimator**	**Confusion effect percentage (%)** **(Eq. **10**)**	**Standard error estimation**	**Lower limit**	**Upper limit**	**Comparison of the standard error of the models with MH (Eq. **11**)**	**BIC**
**Lifetime cocaine consumption prevalence** **(prevalence = 1.8%)**							
PR MH (age adjusted)	2.913	13.08	0.7570	0.786	12.845		
PR Negative Log-Binomial model	2.931	12.38	0.7185	0.717	11.985	5.358	
PR Robust Negative Log-Binomial model	2.931	12.38	0.7144	0.723	11.889	**5.963**	**-43,857.1**
PR Cox/Poisson model	2.928	12.50	0.7161	0.720	11.918	5.711	
PR Cox/ Poisson Robust model	2.928	12.50	0.7152	0.721	11.896	**5.845**	**-43,787.03**
PR Binomial regression	2.925	12.62	0.7128	0.722	11.851	6.201	
PR Robust binomial regression	2.925	12.62	0.7160	0.719	11.903	**5.726**	**-43,602.83**
OR Age adjusted – logistic regression	2.965		0.7184	0.725	12.121		
OR Age adjusted – Robust logistic regression	2.965		0.7198	0.723	12.155		
**Marijuana consumption prevalence** **(prevalence = 9.6%)**							
PR MH (age adjusted)	3.407	2.52	0.3121	1.848	6.281		
PR Negative Log-Binomial model	3.444	1.42	0.3245	1.823	6.506	3.821	
PR Robust Negative Log-Binomial model	3.444	1.42	0.3154	1.856	6.391	**1.046**	**-45,899.8**
PR Cox/Poisson model	3.440	1.54	0.3198	1.838	6.438	2.408	
PR Cox/ Poisson Robust model	3.440	1.54	0.3159	1.852	6.389	**1.203**	**-45,541.37**
PR Binomial regression	3.435	1.69	0.3151	1.852	6.370	0.952	
PR Robust binomial regression	3.435	1.69	0.3164	1.847	6.387	**1.359**	-44,534.91
OR Age adjusted – logistic regression	3.702		0.3246	1.959	6.995		
OR Age adjusted – Robust logistic regression	3.702		0.3255	1.956	7.007		
**Cigarette consumption prevalence** **(prevalence = 21.3%)**							
PR MH (age adjusted)	2.209	17.38	0.1913	1.518	3.214		
PR Negative Log-Binomial model	2.175	19.22	0.2091	1.443	3.276	8.513	
PR Robust Negative Log-Binomial model	2.175	19.22	0.1919	1.493	3.167	**0.313**	**-36,500.12**
PR Cox/Poisson model	2.197	18.02	0.1991	1.487	3.247	3.918	
Cox/Poisson Robust model	2.197	18.02	0.1901	1.863	3.190	0.631	**-35,956.34**
Binomial regression	2.225	16.54	0.1885	1.538	3.220	1.485	
Robust binomial regression	2.225	16.54	0.1878	1.540	3.215	1.864	**-34,252.81**
OR Age adjusted – logistic regression	2.536		0.2105	1.679	3.831		
OR Age adjusted – Robust logistic regression	2.536		0.2127	1.671	3.848		
**Lifetime alcohol consumption prevalence** **(prevalence = 85.7%)**							
PR MH (age adjusted)	1.241	1.13	0.0361	1.157	1.332		
PR Negative Log-Binomial model	1.243	0.97	0.0872	1.048	1.475	58.601	
PR Robust Negative Log-Binomial model	1.243	0.97	0.0360	1.158	1.334	**0.278**	**-45,293.58**
Cox/ Poisson model	1.242	1.05	0.0668	1.089	1.416	45.958	
Cox/Poisson Robust model	1.242	1.05	0.0359	1.157	1.333	0.557	**-44,885.75**
Binomial regression	1.238	1.37	0.0449	1.153	1.329	19.599	
Robust binomial regression	1.238	1.37	0.0450	1.153	1.329	19.778	**-41,961.72**
Age adjusted OR – logistic regression	2.810		0.1260	2.194	3.597		
Age adjusted OR – Robust logistic regression	2.810		0.1248	2.199	3.589		
**Risk of consumption of psychoactive substances (prevalence = 96.1%)**							
PR MH (age adjusted)	1.086	0	0.0185	1.047	1.126		
PR Negative Log-Binomial model	1.086	0	0.0793	0.930	1.269	76.671	
PR Robust Negative Log-Binomial model	1.086	0	0.0186	1.047	1.127	**0.538**	**-47,709.15**
PR Cox/Poisson mode	1.086	0	0.0576	0.970	1.216	67.882	
Cox/Poisson Robust model	1.086	0	0.0186	1.047	1.127	**0.538**	**-47,583.75**
PR Binomial regression	No converge						
PR Robust binomial regression	No converge						
OR Age adjusted OR – logistic regression	3.462		0.1857	2.406	4.982		
OR Age adjusted – Robust logistic regression	3.462		0.1825	*2.421*	4.951		

Models were controlled by grouped age for all cases. Results are shown for the models of negative log-binomial, Cox regression with constant time, log-Poisson, log-binomial compared with MH, and unconditional binary logistic regression model – OR value

The confounding control of the association of depressive symptoms with psychoactive substance use, controlling for age groups, was performed taking as reference the PR of the Mantel and Haenszel (MH) stratification method, comparing with the estimation of the PR of the three models, the differences were less than 1% in the five outcomes, using Eq. 9. The standard errors of estimation of the models showed greater precision when adjusted for robust variance in the three models, showing similar 95% confidence intervals in the five outcomes of psychoactive substance consumption in the three models compared to the 95% CIs using the MH stratification method (Table 2).

### Estimation and precision of the PR with the negative Log-binomial model

The estimated prevalence ratio of the association between depressive symptoms with the different prevalence of psychoactive substance consumption in the study with the negative log-binomial model with and without robust variance adjustment were equal to the crude PR (Table 1).

The prevalence ratio of the association of depressive symptoms controlling for confounding variable by grouped age with the five outcomes showed very high precision with respect to the MH PR, measured with the Eq. 10, with very small percentage of difference of the NLB PR respecting to MH method, being for lifetime cocaine consumption 0.61%, for marijuana 1.07%, for cigarette 1.56%, for lifetime alcohol 0.16% and Risk of consumption of psychoactive substances 0%.

**Fig. 1:**
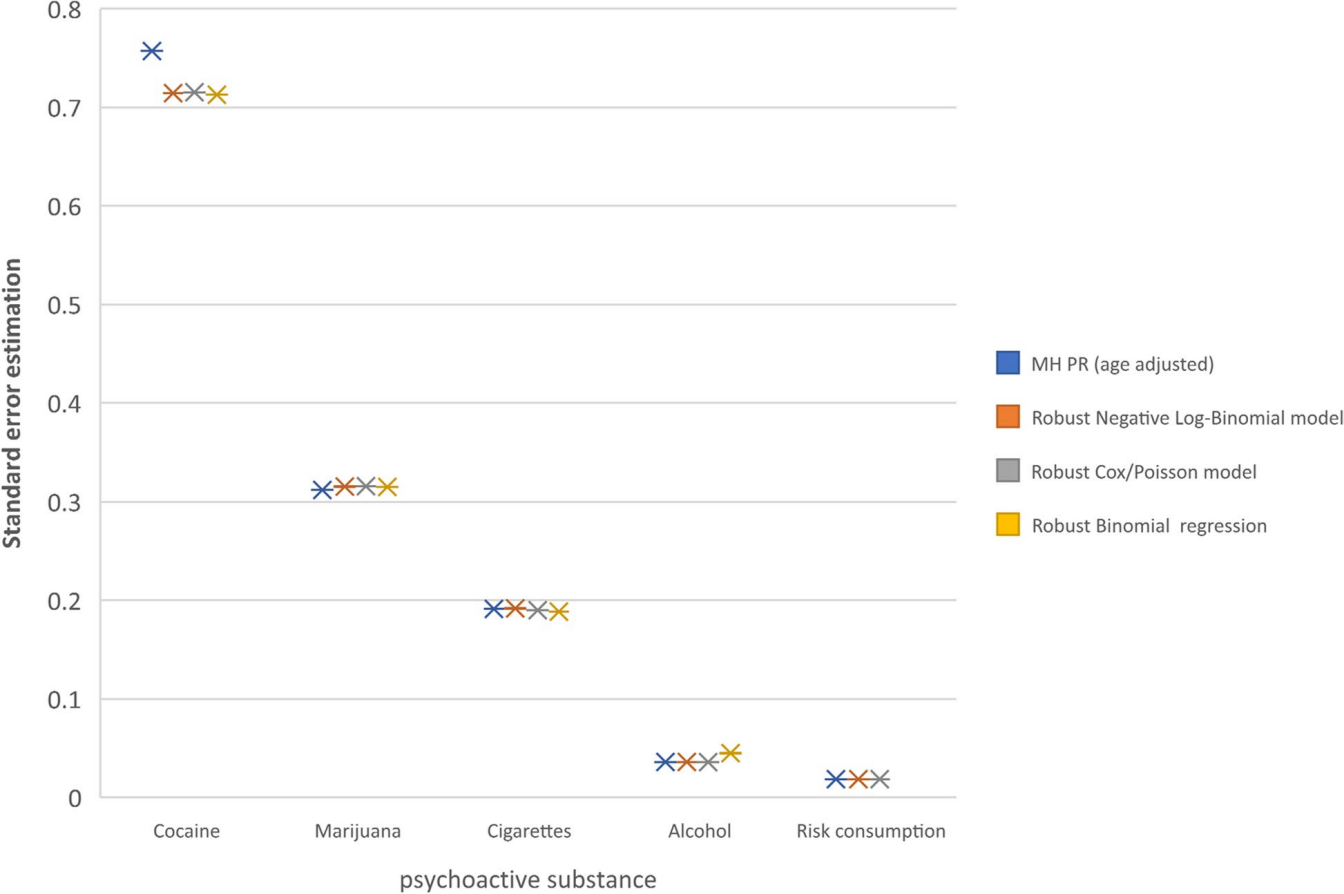
Comparison of the standard errors of estimation of the PR adjusted by age groups, between the models with robust variance and MH

The standard errors of estimation of the NLB model showed greater precision when adjusted with robust variance, showing similar standard errors and 95% confidence intervals for the five outcomes, compared with 95% CIs using the MH stratification method. Using the Bayesian BIC indicator, the NLB showed to be the best model with smaller values than the other models with robust variance. (Fig. 1, Table 2).

The standard errors of PR estimation showed high precision in the robust Cox/Poisson and NLB models with respect to MH, for the 5 consumption outcomes (a small difference in cocaine consumption), with the robust binomial model. showed very large differences in prevalence (85.7% and 96.1%), the standard error of estimation being higher in alcohol consumption and no convergence was found to calculate the risk of consumption of psychoactive substances estimator (Fig. 1).

**Fig. 2:**
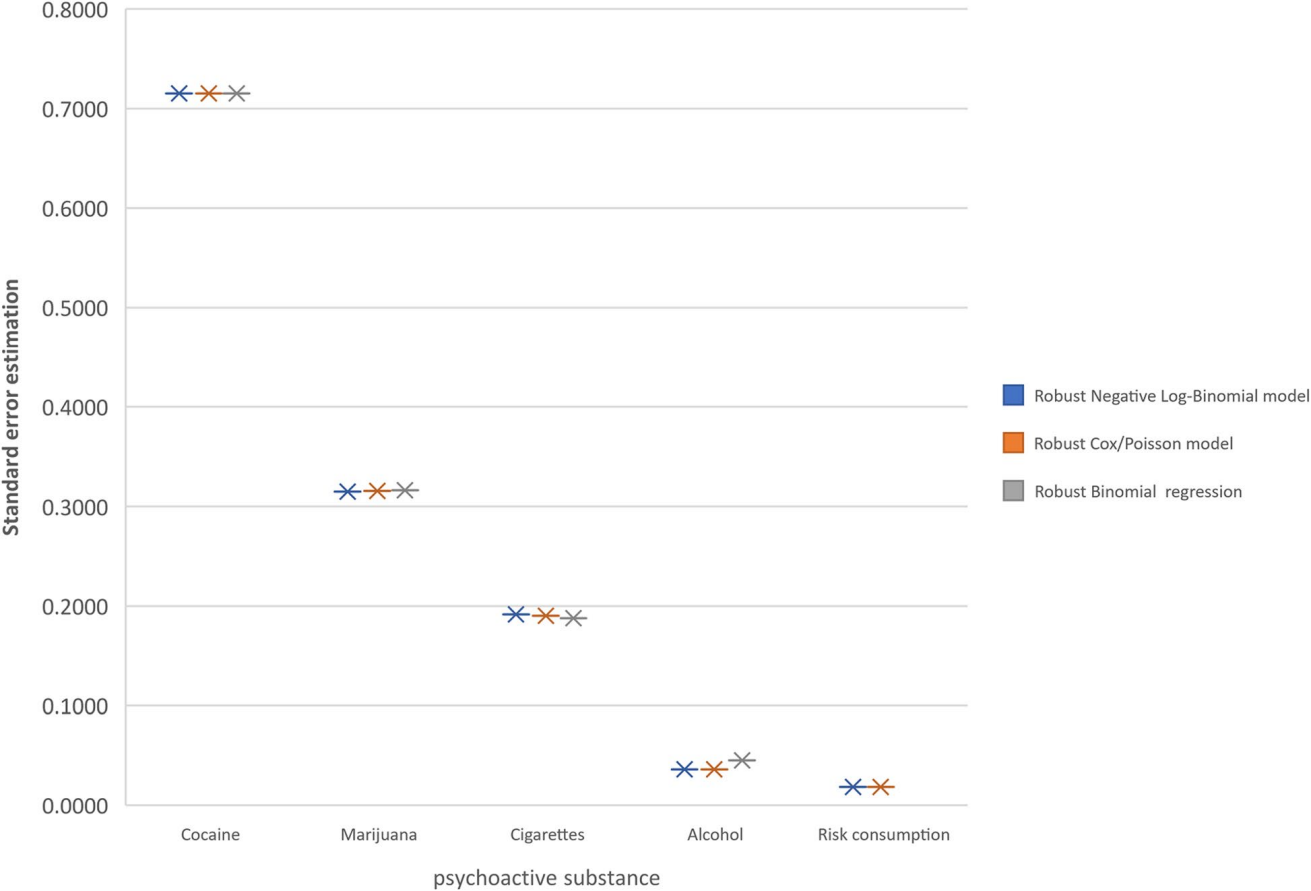
Comparison of the standard errors of estimation of PR adjusted for numerical age, between models with robust variance

In the estimation of the prevalence ratio of the association of depressive symptoms with the five psychoactive substance consumption outcomes, controlling for confounding variable by age measured with a numerical scale with robust variance adjustment, the estimated PR,

the standard errors of estimation and its 95% CI were very similar among the de Cox/Poisson and NLB and in the prevalence of alcohol consumption, which was high (85.7%), the robust log binomial model showed lower precision with a higher standard error of estimate compared to the other models with robust variance and in the highest prevalence of the 5 outcomes (96.1%), no convergence was found to calculate the estimators, neither when age was adjusted categorically, nor numerically (Tables 2 and 3). being higher as the prevalence increases in the psychoactive substance outcomes of the study (Table 3, Fig. 2).

**Table 3:** Comparison of the PR of the models with robust variance and the logistic regression model, for the 5 outcomes, adjusting by numerical age

**Model**		**Cocaine consumption**	**Marijuana consumption**	**Cigarettes consumption**	**Alcohol consumption**	**Risk of consumption of psychoactive substances**
Robust Negative Log-Binomial regression	PR IC 95% See	2.789 (0.873,17.003) 0,7152	3.381 (1.890,6.836) 0.3150	2.168 (1.468,3.342) 0.1915	1.245 (1.050,1.478) 0.0361	1.087 (0.931,1.270) 0.0186
Robust Log Poisson – Cox	PR IC 95% See	2.788 (0.879,16.949) 0,7152	3.371 (1.906,6.767) 0.3156	2.186 (1.512,3.313) 0.1900	1.244 (1.094,1.422) 0.0361	1.087 (0.972,1.219) 0.0186
Robust log-binomial regression	PR IC 95% See	2.787 (0.885,16.894) 0.7152	3.361 (1.921,6.694) 0.3164	2.208 (1.563,3.287) 0.1879	1.245 (1.159,1.337) 0.0452	No converge
Unconditional binomial logistic regression	OR IC 95% See	2.831 (0.690,11.538) 0.719	3.624 (1.917,6.848) 0.325	2.513 (1.664,3.794) 0.210	2.830 (2.212,3.620) 0.126	3.495 (2.430,5.027) 0.185
Robust Unconditional binomial logistic regression	OR IC 95% See	2.965 (0.723,12.155) 0.720	3.702 (1.956,7.007) 0.326	2.536 (1.672,3.848) 0.213	2.810 (2.199,3.589) 0.125	3.462 (2.421,4.951) 0.183

*See* Standard error of estimate

## Discussion

The unconditional binary logistic regression model has been used for the construction of the explanatory associative models of the events of interest in cross-sectional studies of different epidemiological investigations [3–5]. However, this model estimates ORs generating overestimation of the PRs, this bias being greater as the prevalence of the disease or event of interest increases, as was shown in this study in the association between depressive symptoms and the five prevalence of psychoactive substance consumption and risk. The prevalence of psychoactive substance consumption ranged from 1.8% to 96.1%, showing an increment in the inherent bias in the association between depressive symptoms and psychoactive substance consumption as the prevalence of intake increased.

In estimated prevalence less than 10%, it is expected that the inherent overestimation bias of OR versus PR was minimal in the cases of smaller prevalence of the disease [6]. In this study it was shown with the prevalence of cocaine consumption which was 1.8%, which was associated with depressive symptoms, the estimates were very similar between OR and PR (OR = 3. 337, 95%CI: 0.819,13.600 vs. PR = 3.294, 95%CI: 0.815 vs. 13.315), and when controlling for confounding factor by grouped age with the NLB model with robust variance and with MH (OR = 2.965, 95%CI: 0.725, 12.121 vs. PR = 2.931, 95%CI: 0.723, 11.889) and was very accurate controlling for numerical age as shown in Table 3.

In studies since 2003 and 2008, several explanatory models were proposed for the estimation of the PR and solved the overestimation of the OR of binary logistic regression models [3, 4]. Among the analyzed models, Cox constant time model of regression for proportional bias and two generalized linear models (Poisson regression and Log-binomial regression) were proposed by the authors using applied research examples carried out in STATA version 7.0 and 9.0. Thus, in order to compare our proposed method of NLB, we included these three previous models for the estimation of PRs an 95% CIs with and without robust variance, as a validation for our results with the NLB model. In previous studies, Cox constant time model, Log-Poisson regression, and Log-binomial regression were used to estimate PRs, giving the same results as those obtained for the crude PR and 95% CI in 2 × 2 tables and good accuracy in controlling for confounding variables when compared to PRs obtained with the MH stratification method [3, 4].

In this study we found concordant results with the 2003 and 2008 studies, in the crude estimates and in the control of confounding variables with the PR and their 95% CIs, as well as in the respective standard errors, in which the joint conclusion of these studies is that Cox constant time model and Log-Poisson regression with robust variance give as a result really accurate estimates for the PRs and its standard errors for the estimations in cross-sectional studies [3, 4, 13–17], whereas the Log- binomial model without robust variance adjustment showed accurate estimates at intermediate and even small prevalence [17–19], as it was visualized with our results for the case of cocaine and marijuana consumption. However, it is important to highlight that for the case of the Log-binomial model adjusted with robust variance in small prevalence < 10%, such as for cocaine and marijuana consumption, the standard errors were larger than those without adjustment, in contrast for what it was expected with the adjustment of robust variance for the three models, including the proposed negative log binomial model additionally, this model showed greater precision with lower estimation standard errors with respect to MH, than the other models with robust variance.

In the previous studies, issues related with the estimations and mathematical models were identified for the three methods. In the Log-binomial generalized linear model, due to non-convergence in the outcomes with very high prevalence, outcomes in the estimates of the PR and in its standard errors for this GLM model are performed with the maximum likelihood estimation method using iterative methods to reach estimates with convergence tolerances with epsilon (ε) less than 0.000001. In our study, the log binomial model with robust variance estimated a very high standard error compared to high prevalence such as alcohol, compared to the Cox/Poisson and NLB models with robust variance, and without convergence to obtain the PR estimators, for the risk of consumption of psychoactive substances, which has a high prevalence of 96.1%.Also, the non-convergence in the numerical confounding variables was solved, controlled with the abovementioned estimation and optimization methods, available in the statistical software such as STATA version 15 and later, in high prevalence as in our study that found convergence for the prevalence of alcohol of 85.7%, although this was not the case for very high prevalence, such as the risk of consumption of psychoactive substances of 96.1%, which did not generate convergence to obtain the estimates of the PR [11].

For the overdispersion (extra-Poisson variance), the adjustment with robust variance proposed by Lin and Wei [18] was the alternative for the issues in the Poisson model for the cross-sectional studies as it was shown previously [3, 4]. However, even after the adjustment of the estimates of the Poisson model with the robust variance, it still shows low efficiency with a higher sampling variability than that required for the model estimators [19]. In our study it was observed that using the Log-Poisson model with robust variance it was found an adjustment in the accuracy of the standard errors and therefore to the 95% CI, finding the same results as in the Cox constant time regression model with robust variance adjustment, which were very concordant with the NLB and log-binomial model with robust variance for the estimation of PRs in prevalence that are not high, in cross-sectional studies.

The negative Log-binomial (NLB) regression model, which was the GLM proposed model in this study, showed accuracy with respect to the crude values of PR, standard errors and 95% CI, with the adjustment of robust variance for the standard errors’ estimations. In the case of adjustment and control of confounding variable of grouped age, results showed a very high precision with those obtained with the MH stratification method, as well as for the estimations of PR, standard errors and 95% CI with the Cox constant time model, Log-Poisson, and Log-binomial regression models when adjusted with robust variance. Very concordant results were obtained for the associations between depressive symptoms with the five psychoactive substances consumption, controlling for the numerical age, for the estimates obtained with the three available models with the adjustment for robust variance.

The NLB model with robust variance adjustment is optimal in epidemiological cross-sectional studies through surveys and in some cases with clinical approaches, to estimate the prevalence of a particular disease or event of interest and its associated factors through the multivariate NLB model with robust variance adjustment, without bias of overestimation in the associations and with high accuracy for the estimates. It allows the construction of associative models to identify the groups with the highest risk of the disease or event of interest within a population. The use of NLB model could also be applied for the development of prevention and control programs for specific conditions, and in the future to decrease the prevalence of those particular conditions. Also, it could impact policy makers and decision-making for the control or follow-up of the conditions of interest within a population with high prevalence values.

## Conclusions

The negative log-binomial generalized linear model with robust variance is an optimal multivariate model for the construction of explanatory associated factors of disease or binary events of interest in cross-sectional studies, generating estimates with very high precision of the prevalence ratio, standard errors of estimation and confidence intervals, when adjusting for categorical and numerical confounding variables. This NLB model is mathematically constructed to identify the variance that could not be explained by the Poisson model in cases of overdispersion, and thus, NLB model is proposed as an alternative for those cases of overdispersion. Finally, the NLB model does not present convergence issues in the estimates of the PR and the standard errors of PR estimation, in large or small prevalence in cross-sectional studies. NLB model is proposed as a novel alternative for the analyses of prevalence ratios in cross-sectional studies, independent of high or low prevalence of the disease or the event of concern and with greater precision than the other models with robust variance with respect to MH and with the BIC Bayesian indicator, as it was shown in the current data set of this cross-sectional study.

## Data Availability

The data of the database used is available under request to the authors (basefinal.sav) due to confidentiality issues.

## Abbreviations

CI: Confidence interval
GLM: Generalized linear model.
IRLS: Iteratively reweighted least squares
IRR: Incidence rate ratio
MH: Mantel Haenszel stratification model
NLB: Negative Log-binomial regression model
OR: Odds ratio
PR: Prevalence ratio
